# Components wear detection by piezo-acoustic sensors and influence on the material in progressive stamping

**DOI:** 10.1038/s41598-026-54089-9

**Published:** 2026-05-19

**Authors:** Juras Skardžius, Justinas Gargasas

**Affiliations:** Vilnius Gediminas Technical University, Saulėtekio al. 11, Vilnius, 10223 Lithuania

**Keywords:** Stamping, Progressive tool, Wear, Piezo-acoustic sensor, Real-time monitoring, Material deformation, Engineering, Materials science

## Abstract

Progressive stamping is widely used for high-volume sheet metal production, where tool condition directly affects stability and product quality. Tool wear, such as punch and die edge rounding, impacts the piercing mechanism and increases process forces and burr formation, and causes part edge deterioration. Therefore, accurate real-time wear monitoring is essential to prevent failures and reduce downtime. This study explores piezo-acoustic sensors for real-time monitoring of punch wear. Experiments on DC01 steel included static and dynamic tests with predefined tool wear. Sensor signals and force data were continuously analyzed. Results show that increased wear leads to higher peak load and delayed material fracture, indicating a shift from shear-dominated piercing to deformation-dominant material separation. Microstructural analysis confirmed that wear significantly alters fracture morphology and subsurface deformation, impacting part quality. Research shows that piezo–acoustic sensing yields the peak load value for punch wear, making it a powerful and useful measure in industry. It provides an effective solution for real-time tool-condition monitoring and predictive maintenance in progressive stamping operations.

## Introduction

Tool wear is critical to any sheet metal stamping and forming operations because it directly impacts process stability, part quality, and operating costs. In progressive and other high‑volume stamping processes, the interplay between the punches and dies in the tool and the workpiece involves repeated high‑pressure contact, sliding, and plastic deformation, progressively deteriorating the working elements’ surface geometry and the tool’s overall wear.

Wear modifies contact conditions and increases the amount of forming force, which can contribute to dimensional discrepancies, burr formation, and surface defects in production. The key types of tool wear in stamping are abrasive and adhesive wear, galling, and fatigue. Abrasive wear occurs when hard asperities or debris embedded in the contact interface plough or micro‑cut the tool surface, thereby scratching or removing material by abrasive wear^1^.

Abrasive wear is one of the major factors contributing to the degradation of progressive stamping tools, especially under high contact pressure and repeated sliding. It is a result of hard aspirations on the sheet material, or entrapped wear debris serving as micro-cutting implements, which penetrate and remove material from punch and die surfaces via ploughing and micro-machining. The latter effect is enhanced in progressive stamping by cyclic contact and the continuous generation of metallic particles, which are trapped at the tool-workpiece interface, thereby accelerating three-body abrasion. Abrasive wear is strongly dependent on variations in material hardness, surface roughness, and lubrication effectiveness; the lack of lubrication promotes direct metal-to-metal contact, leading to more severe surface damage^2^. The abrasive wear on these surfaces is concentrated in those parts having the greatest contact stresses and the largest sliding distances. Here, the cumulative effect of material removal results in edge rounding, loss of dimensional accuracy, and increased forming forces, which eventually lead to quality loss and promote wear mechanisms such as adhesion or fatigue^3^.

Adhesive wear and galling occur when high contact pressures and friction induce local micro‑welding and material transfer between the workpiece and tool surfaces, thereby degrading the tool profile and favoring further damage during ongoing production^4^. These mechanisms are not mutually exclusive; on the contrary, they can work in conjunction or switch depending on the contact pressures, material types, sliding distances, and lubrication conditions involved. Experiments show that tool wear is less evenly distributed across the tool surface and more localized in critical areas, such as the corner radius of the dies, where contact pressures and sliding distances are high and rapidly change over the cycle. In those areas, ploughing mechanisms are more predominant in inner parts, whereas galling and adhesive transfer are prevalent at transient contact points, and the early contact stages are of utmost importance for a tool’s life^5,6^.

In progressive stamping processes, another important degradation mechanism is fatigue wear, owing to the cyclical nature of the loading experienced by punches and dies. Every stroke induces repeated high contact stresses, and microcracks are initiated at or below the tool surface, especially at cutting edges and the die radius. These cracks, over time, propagate under cyclic loading conditions and ultimately result in material removal via either pitting, spalling, or surface delamination. Fatigue wear is especially high in high-volume production, with tools reaching millions of cycles, and it is exacerbated by stress concentrations, residual stresses, and fluctuating contact conditions during piercing. In progressive stamping, the mechanism typically occurs alongside abrasive and adhesive wear, as crack-induced surface damage exposes fresh material that is more susceptible to further wear, ultimately accelerating tool degradation and reducing tool life^7^.

Contact geometry significantly affects where and how wear occurs. Alterations in the die radius profile could result, even at small scales of tens of micrometers, in a remarkable transformation in local contact pressure distributions and wear severity. Experiments have shown that fine control of die profile geometry can prolong tool life by reducing peak contact stresses and redistributing wear over a larger area^8^.

Material properties make the wear behavior even more complicated. The increasing prevalence of advanced high‑strength steels (AHSS) in automotive and aerospace applications intensifies wear forces caused by higher flow stresses and harder microstructural constituents, raising contact pressures and frictional interactions. In the case of AHSS and coated steels (e.g., Al-Si-coated press‑hardening steels), debris compaction and mixed wear regimes take over, including adhesive material transfer and third‑body abrasive action by fractured coating particles during compaction and mixed wear modes, resulting in faster wear progression. In forming tools, wear progression is inherently non‑linear as well^9^.

Research in fine blanking and forming^10^ has shown that wear may progress through several stages: an initial running‑in phase, during which surface degradation occurs due to abrasive and micro‑cutting effects at the onset; a stable, adhesive‑dominant stage; and an accelerating phase, when wear leads to fatigue cracking and functional failure. This three‑stage behavior clearly illustrates that early contact conditions and material behavior play a vital role in cumulative wear and tool life^11,12^.

Friction and lubrication are just as important in wear behavior. Lubricants prevent direct metal-on-metal contact, decreasing the friction coefficient and preventing galling; however, in high‑strength steels and high‑speed stamping, lubricant films are known to fail or not be properly maintained, increasing adhesive and abrasive action. Sliding contact at high pressures can also generate local heating, which has a negative effect on friction in the region and consequently on the wear rates. In conclusion, tool wear in stamping and forming is a complex tribological property influenced by material properties, contact geometry, loading conditions, and the efficacy of tool-controlled lubrication. These factors interact to create a range of mechanisms of wear - abrasive scuffing, adhesive galling, fatigue cracking, and mixed regimes - that ultimately decide tool performance and part quality. Being familiar with these mechanisms and their progression is critical for enabling reliable monitoring, selecting suitable tool materials and coatings, and optimizing process parameters to maximize tool life and production performance.

Monitoring the overall pressing force (tonnage) has long been one of the most widely used indirect metrics for in-process condition monitoring in sheet metal stamping. Tonnage in a stamping press is the force applied to the tooling and workpiece in its forming cycle by the ram (in accordance with strain sensors on the press frame, tie rods, or columns). This force signal creates a time-dependent load profile that represents the integrated mechanical response of the workpieces during deformation and the tool–material interaction during the cycle^13^.

Wear and tool anomalies modify the mechanical resistance faced during forming, the fundamental rationale for force/tonnage monitoring. As punch-and-die edges become rounded by progressive wear, frictional resistance and plastic deformation increase during piercing or bending operations, resulting in measured increases in peak force and changes in the total shape of the force–time curve^14^. In classical monitoring approaches, peak tonnage or global area per unit of force have been used qualitatively as parameters to gauge wear or pressing anomalies, such as misalignment, insufficient lubrication, or material thickness variation. These trends arise from the fact that worn tooling generally requires greater deformation force than a sharp tool, since deformation (detailing the separation of material) arises from forming forces^15^.

Although tonnage accounts for the global reactive load of the press, it remains a compound signal and comprises the influences of all tools operating simultaneously and all stages in a forming cycle (e.g., blanking, bending, drawing). This complicates separating the impact of a single tool’s wear from the global force profile, especially for multi-station progressive dies, where dozens of working cutting and forming elements operate in a single stroke. It has been highlighted in recent research on progressive die stamping that total tonnage variations might not just be due to tool wear, but rather there may be the presence of various interacting factors that impact the results such as bottom dead center (BDC), sheet thickness variation, lubrication state, and press dynamics, making it challenging for one to interpret tonnage data in any simple manner^16^.

One major drawback of conventional tonnage monitoring is its poor spatial resolution. Due to the strain-based tonnage sensor’s ability to quantify elastic deformation forces in the press layout (column bending), the signal corresponds to network activity across all contact points of the active tool-workpiece, rather than localized connections at the punch edge or die edge. Hence, wear induced at one level of the work process can be concealed by the dominant force effects of other operations in the cycle, especially in complex progressive stamping, where several cuts and forming stations combine to affect the overall load^15,17^.

Also, variability in the stamping process and noise can hinder estimation performance. Tonnage profiles can also differ dramatically across strokes, as unavoidable material properties, lubrication parameters, strip tension, and physical machine behavior may not all be constant even in the absence of major tool wear. This variability can affect the signal-to-noise ratio and yield higher false alarms in monitoring systems that use simple thresholding on peak forces or tonnage values^18^.

To address these challenges, the authors have explored force segmentation techniques and localized load-cell strategies to decompose the total tonnage signal into subcomponents corresponding to specific tool behaviors. Conversely, an important concept is in-die force sensing, in which force sensors can be inserted into the tooling (e.g., in die holders) to record contact pressure over time at the site, each of which is characteristic of a single station. In doing so, a spatial map of forming pressures is laid out, usually based on numerical interpolation (e.g., Thin Plate Splines or Bezier surfaces) to develop a more detailed picture of the tool–workpiece interaction at the local scale. This method improves observability of an important event like misalignment or premature wear (such as misalignment) at the edges of particular tools^19^.

Sah and Gao^14^ demonstrated that, if contact pressure is sensed by spatially distributed sensors in tooling and interpolated across the workpiece interface, a temporal–spatial contact pressure surface can be reconstructed that more coherently reflects forming dynamics than global tonnage alone. Their work makes the point that while tonnage provides useful global information for our purposes, fine-grained force measurements are necessary to assess tool conditions precisely. Sensing strategies using embedded devices can detect small anomalies such as slide mis-parallelism and local deformation that aren’t easily detected by tonnage alone^15^.

Still, local measurements are difficult to put into an industrial context. Embedding sensors in tooling adds complexity to the system, necessitates ruggedization to survive the harsh stamping environment, and increases the data’s dimensionality. This frequently results in a trade-off between sensor accuracy and practicality, especially for progressive tools with tens of stations. Researchers continue to explore hybrid strategies that combine tonnage, localized force, and other sensor modalities (e.g., acoustic emission, vibration) with advanced data processing to improve monitoring performance.

Advanced signal processing techniques for extracting useful features from force/tonnage profiles are not widely known among many sensors these days. The wavelet transforms, recurrence plots, and statistical pattern recognition based on wavelet analysis of tonnage signals are some of those techniques applied to the tonnage signal to slice cycles, isolate the phases with respect to parts of the process (for example, piercing and bending), and analyze to detect subtle changes concerning tool wear or process anomalies. Wavelet analysis, among others, can help discriminate between normal shape variation (which could be due to material heterogeneity) and feature changes due to tool wear by segmenting signals into time-frequency elements, thereby separating normal shape variations from changes due to tool wear^20,21^. While these approaches increase the interpretability of tonnage results, they also illustrate the difficulty of directly associating tonnage data with global force data alone for multi-operation processes. The shift toward smart manufacturing, which is central to the Industry 4.0 paradigm, underscores this need. Industry 4.0 aims to establish fully autonomous, interconnected production systems where data-driven decisions optimize every facet of the manufacturing process. In this light, automatic fault checking and predictive maintenance are not nice to have but are essential components. As one study notes, the development of new and more complex tools and processes, as well as the required quality standards, necessitates that 100% inspection become the standard, making automated in-process monitoring crucial for the forming sector. Robust Tool Condition Monitoring (TCM) systems are the enabling technology driving this transformation, promising to reduce costs, enhance productivity, and ensure process stability.

In contrast to conventional tonnage-based monitoring, which shows the overall reaction of the press, the proposed technique integrates piezo-acoustic sensing localization within the tooling environment. This allows increased sensitivity to changes in individual tool condition, particularly at the punch level. In addition, the study’s unified framework employs controlled quasi-static experimentation, real-world production validation, and microstructural analysis. This multi-scale method not only enables detection but also gives a physical interpretation of wear-induced variations in the mechanisms of deformation. In this context, the novelty of this work is:

1. Piezo-acoustic sensing for localized wear detection in progressive stamping;
2. Validation of peak load as a robust industrial feature under varying loading regimes;
3. The relationship between sensor signals and microstructural evolution.

## Experimental research methodology

Research was performed in two main steps: (1) Tests were taken under fully controlled conditions with a tensile machine in a laboratory in order to assess the general punch wear model (static tests); (2) Stamping tests were conducted under controlled conditions in real-life conditions (dynamic tests).

**Fig. 1:**
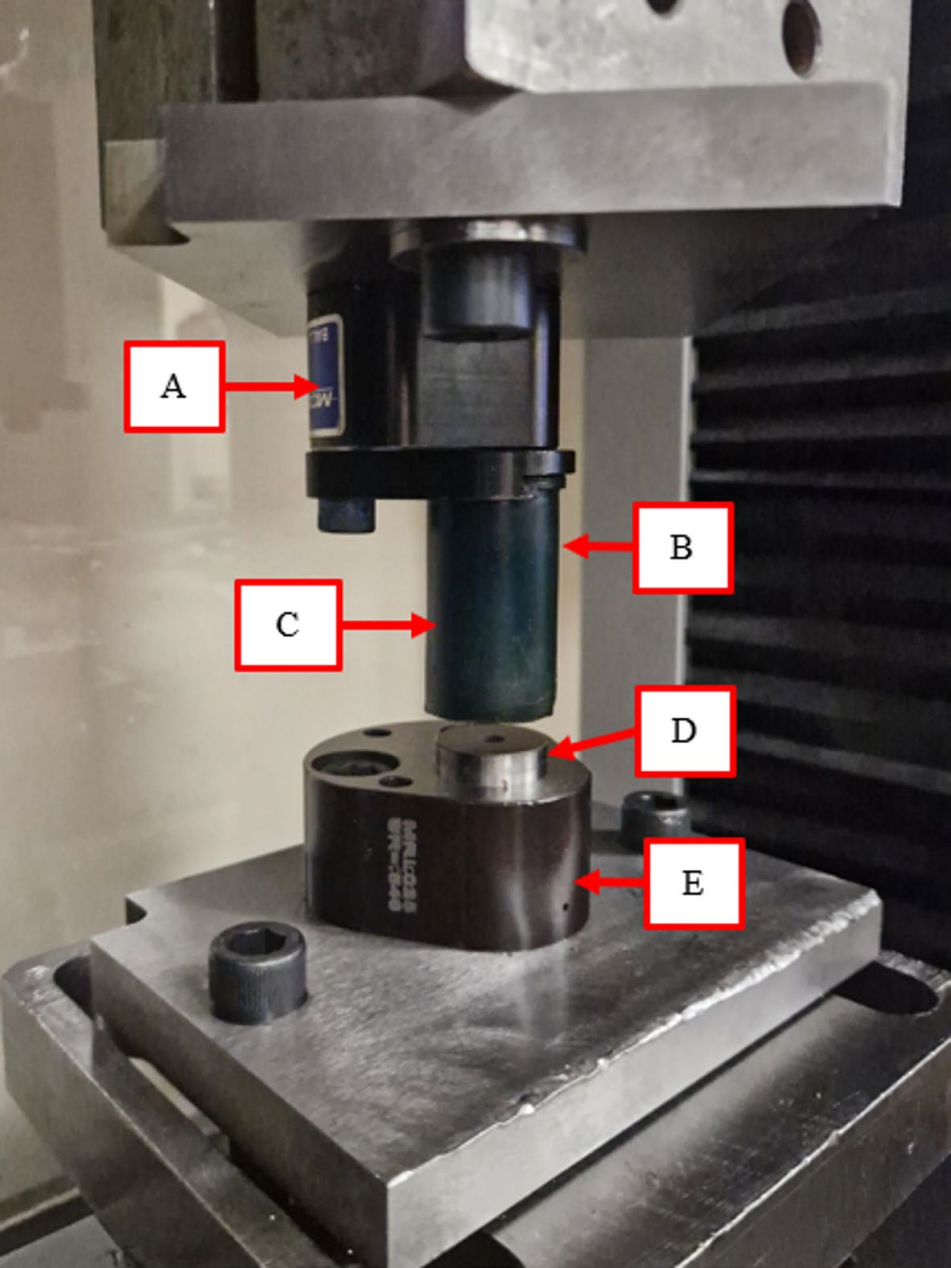
Set-up for test in tensile machine: (a) Punch holder, (b) stripper; (c) punch inside of the stripper; (d) die; (e) die holder.

Static or fully controlled tests were performed with a tensile machine – Zwick BZ2-MMAG100.SH01 in laboratory appropriate conditions. Technical specifications are presented in Table 1, and the test setup is presented in Fig. 1.

**Table 1:** Technical data of the tensile machine.

Characteristic	Value
Manufacturer	Zwick GmbH & Co. KG
Model	BZ2-MMAG100.SH01
Maximum test load	100 kN
Positioning repeatability	± 2.0 μm

While performing tests with the tensile machine, an attempt was made to reproduce real-life test conditions as accurately as possible, but due to the machine’s mechanical limitations, it was impossible to match the same cycle speed. Test parameters are presented in Table 2.

**Table 2:** Test parameters.

Characteristic	Value
Pre-load	50 N
Test speed	0.4 mm/s
Tool separation	15 mm
Distance till billet	9 mm

Dynamic or real-life tests were performed using the automatic press “SMV 106T”, operated at a constant speed of 40 cycles per minute, with lubrication levels set according to the specifications outlined in the tool’s instructions (6 g/m^2^).

Stamping test data was acquired from the flywheel angle sensor (CEV58S-00117) and the piezoelectric sensors installed in the tool. The technical specifications for the shaft position sensor are detailed in Table 3, and for the piezoelectric disc in Table 4. The signal processing for the piezoelectric sensor is performed using the proprietary UNiDOR compactPRESS smartLINE system. The internal processing algorithms (e.g., filtering and signal conditioning) are not directly accessible due to industrial system constraints; therefore, the key acquisition parameters relevant to this study are defined at the system interface level. The signal was acquired at a high sampling rate synchronized with the press-angle signal, ensuring sufficient temporal resolution to capture the rapid piercing event (~ 0.005–0.006 s). Built-in noise filtering and signal conditioning optimized for stamping applications were implemented in the system. In this study, the extracted feature was the peak value within each cycle, identified directly from the processed sensor output. This method aligns with common industrial practice, which prefers robust, computationally efficient features over complex signal decomposition. This approach thus guarantees reproducibility at the application level while still incorporating low-level signal processing into the industrial monitoring system. To address process variability, statistical representation of the results was added in the form of value distribution plots and summarized dispersion indicators. These additions provide insight into repeatability and confirm that the observed trends are not due to random fluctuations.

**Table 3:** Technical specification of the shaft position sensor.

Characteristic	Value
Manufacturer	TRsystems GmbH, Division Unidor
Model	CEV58S–00117
Resolution	≤ 33 bits
Measurement count	≤ 256,000

**Table 4:** Technical specification of the piezoelectric disc sensor.

Characteristic	Value
Manufacturer	TRsystems GmbH, Division Unidor
Model	PSA 10.5
Capacity	9000-12000pF
Resonance frequency	> 6.4 kHz
Impedance	200–700Ω

**Fig. 2:**
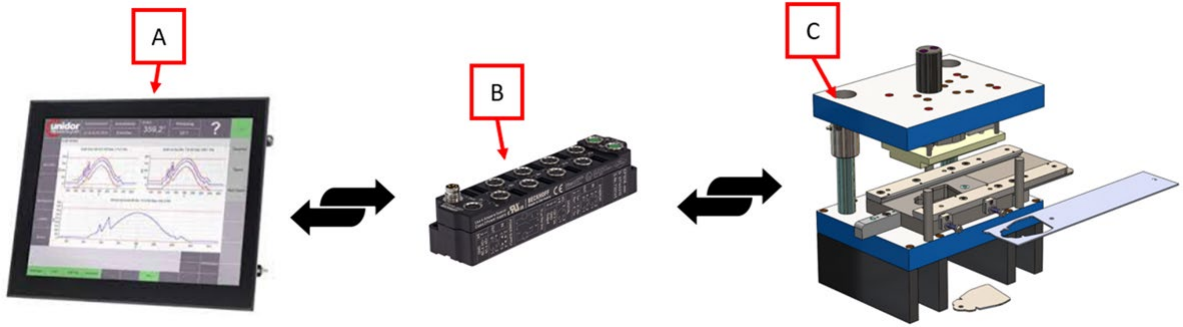
The communication setup of the sensor system: (a) a display interface and control unit, (b) a connection adapter, and (c) the sensor modules.

The mentioned sensors were interfaced with the compactPRESS smartLINE data-processing and control system. At the core of this system is an intelligent supervisory controller that continuously monitors and evaluates incoming sensor signals. This functionality enables effective protection of both the press and associated tooling while ensuring stable, efficient production operations. The remaining system components are summarized in Table 5, and their integration and functional roles within the stamping line are illustrated in Fig. 2.

**Table 5:** Technical data of the data processing system.

Characteristic	Value
Manufacturer	TRsystems GmbH, Division Unidor
Model	UNiDOR compactPRESS smartLINE
Controller	Ether CAT Master RT
Press angle setting module	CEVV58S–00117
Analog signal input module	Ether CAT Box

**Fig. 3:**
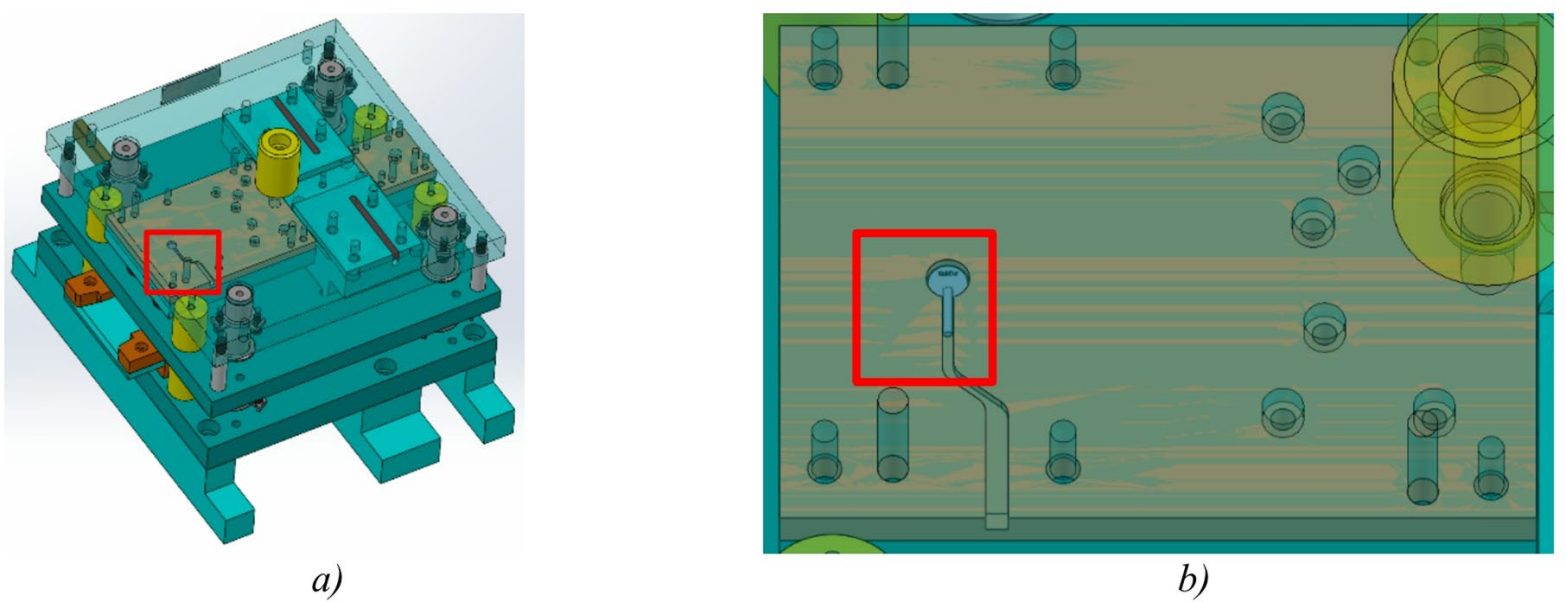
The position of the piezoelectric disc sensor is shown in two perspectives: (a) a model view and (b) a direct model view.

A piezoelectric sensor was placed directly above the test punch to obtain the best possible readings. Sensor installation location is illustrated in Fig. 3.

**Fig. 4:**
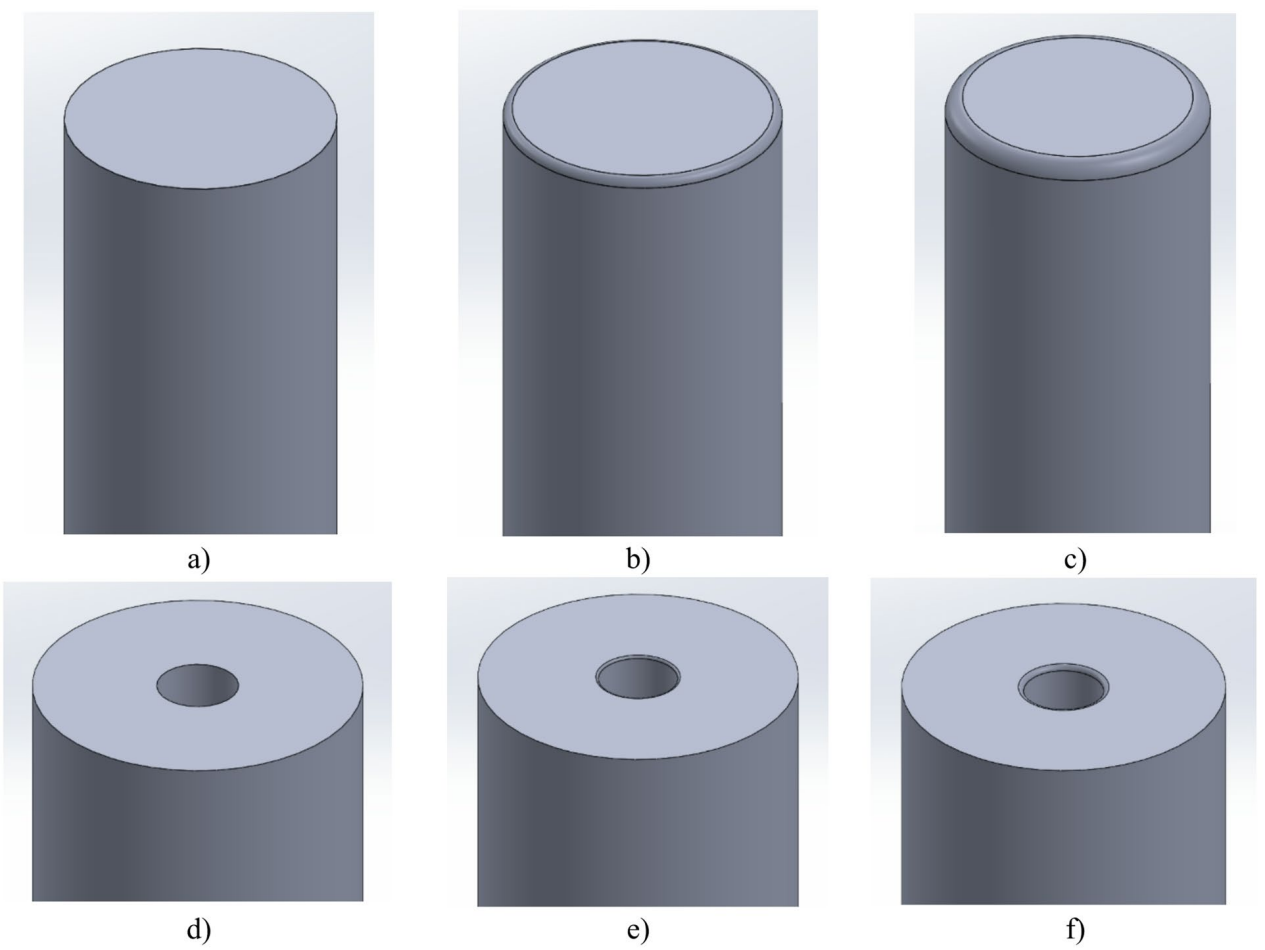
Punch and die with radii used for tests: (a) Punch radius R0.0; (b) Punch radius R0.2; (c) Punch radius R0.4; (d) Die radius R0.0; (e) Die radius R0.2; (f) Die radius R0.4.

Both experiments were carried out using the same DC01 steel sheets, with a nominal thickness of 2 ± 0.02 mm, and the same punches and die sets. To simulate wear during tensile machine testing, a punch-and-die set (Fig. 4) with factory-prepared radii on the edges of the working area was used. Such an approach provides a simplified geometric estimation of degradation. However, it does not fully reflect the complexity of real-world wear mechanisms. In reality, the wear process involves abrasive, adhesive, chipping, and plastic processes that lead to wear with irregular edge shapes and local damage, rather than uniform rounding. Although the assumption of a round edge allows for parametric study under controlled conditions and approximates the trend in edge bluntness fairly, it inevitably overlooks changes in microstructure, surface roughness, and non-uniform wear characteristics. It should be noted that such an approach should be regarded as a theoretical estimation, which reflects primary factors but ignores secondary ones. Therefore, the results should be interpreted purely in terms of the geometry-driven process sensitivity, rather than as a model predicting the tool lifetime or the wear progression. Note that this study does not extrapolate beyond edge radius effects to industrial wear behavior. However, this controlled approach provides a well-defined baseline for quantifying the first-order influence of tool edge geometry on process response. The tests and combinations used in the research are presented in Table 6.

**Table 6:** Combinations of set’s.

Combination No.	Group No.	Punch radius, mm			Die radius, mm		
		R0.0	R0.2	R0.4	R0.0	R0.2	R0.4
1	1	X			X		
2		X				X	
3		X					X
4	2		X		X		
5			X			X	
6			X				X
7	3			X	X		
8				X		X	
9				X			X

After the various tests, the representative combinations corresponding to low, intermediate, and high wear were chosen for scanning electron microscope (SEM) microstructural evaluation. SEM analysis affords high spatial resolution up to ~ 10 nm, allowing detailed evaluation of microstructural evolution, grain morphology growth, and the initiation/ propagation of defects. Cross-sectional micrographs were obtained by acquiring partially overlapping images in consecutive sequences from focused regions of the specimens, with an overlap ratio from 20% to 45% to guarantee continuity and reconstruction of the areas studied. As the specimens were made of ferromagnetic materials, the interactions between the incident electron beam and the induced magnetic field produced additional contrast effects, enabling qualitative analysis of material composition and contributing to a more comprehensive interpretation of the effects of experimental variables. The effects of wear on the subsurface structural integrity and overall material quality were further investigated by analyzing residual material and wear debris to understand the mechanisms by which subsurface damage develops and degrades. No quantitative measurements of deformation layer thickness or shear band width were performed in this study.

## Results and discussion

### Wear effects on signal recognition

**Fig. 5:**
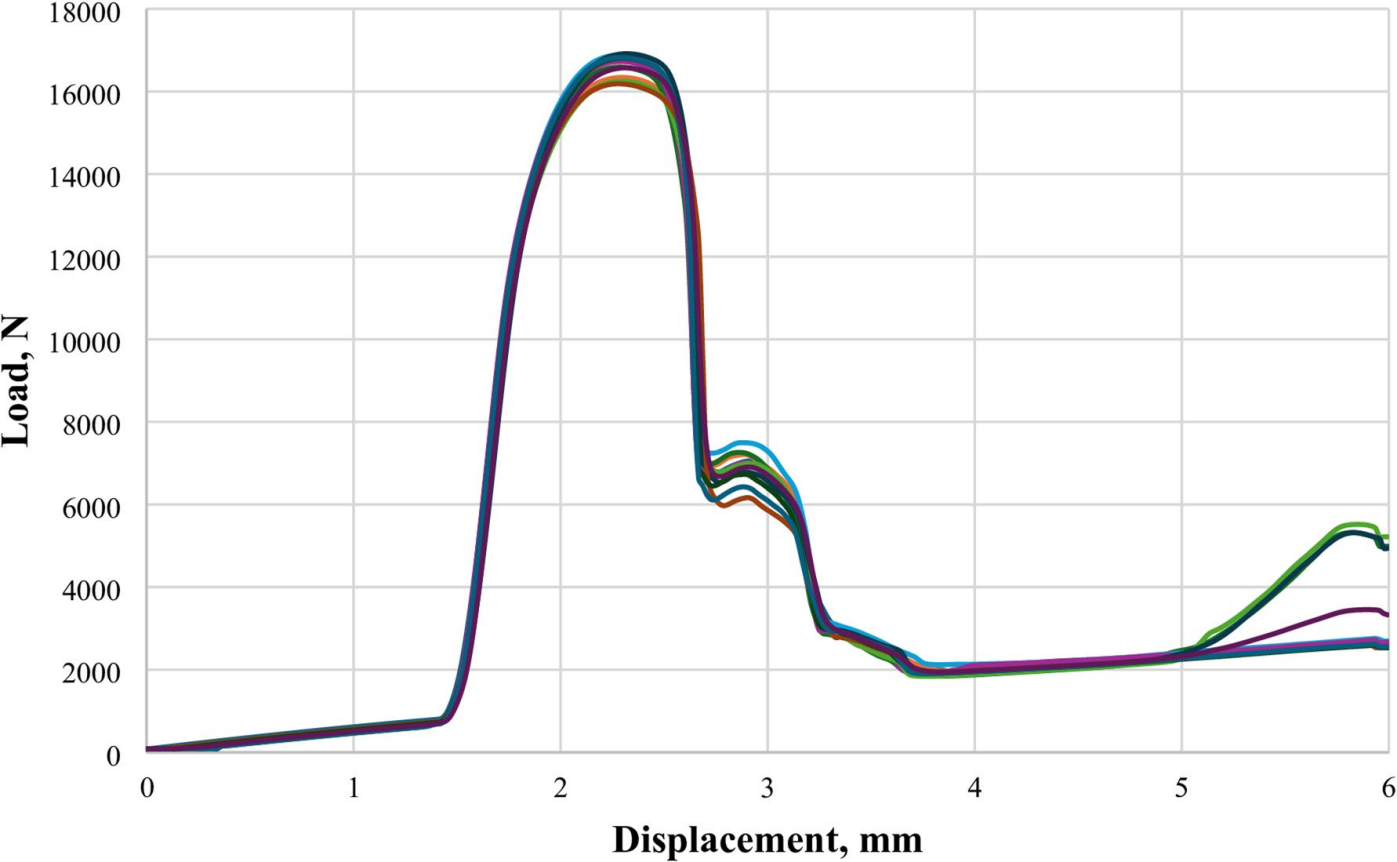
First combination, 10 repetition graphs.

**Fig. 6:**
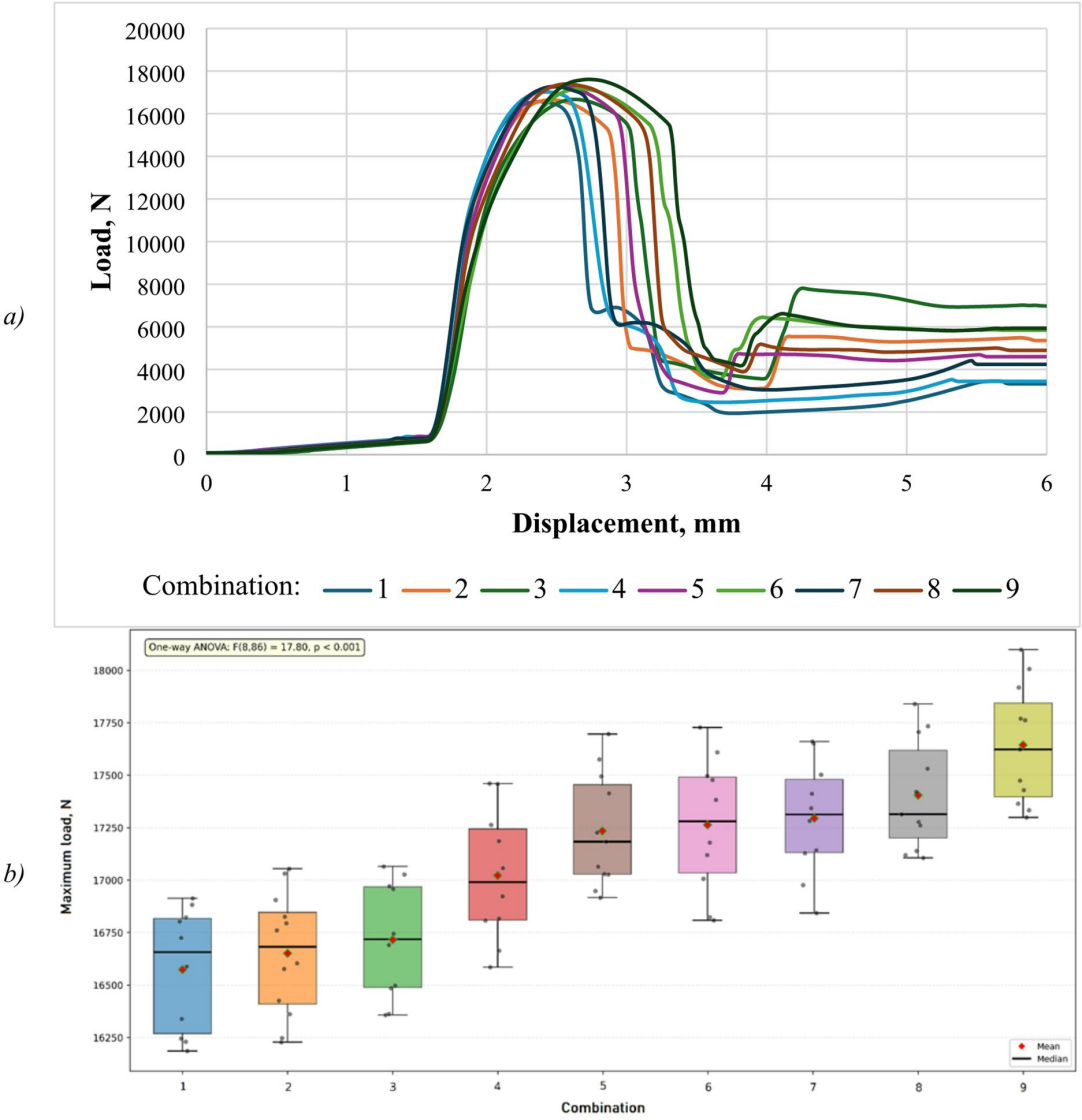
Static tests average load values comparison: (a) Graph view; (b) Recorded values distribution view.

The study started with static tests using a tensile machine. With the setup noted in Fig. 4 and the parameters noted in Table 5, 10 repetitions were performed for each combination mentioned in Table 6. After 10 repetitions, the average value graph was created from the gathered data and used for further investigation. A sample of gathered 10-repetition data is presented in Fig. 5, and all 9 combination average graphs are presented in Fig. 6.

**Fig. 7:**
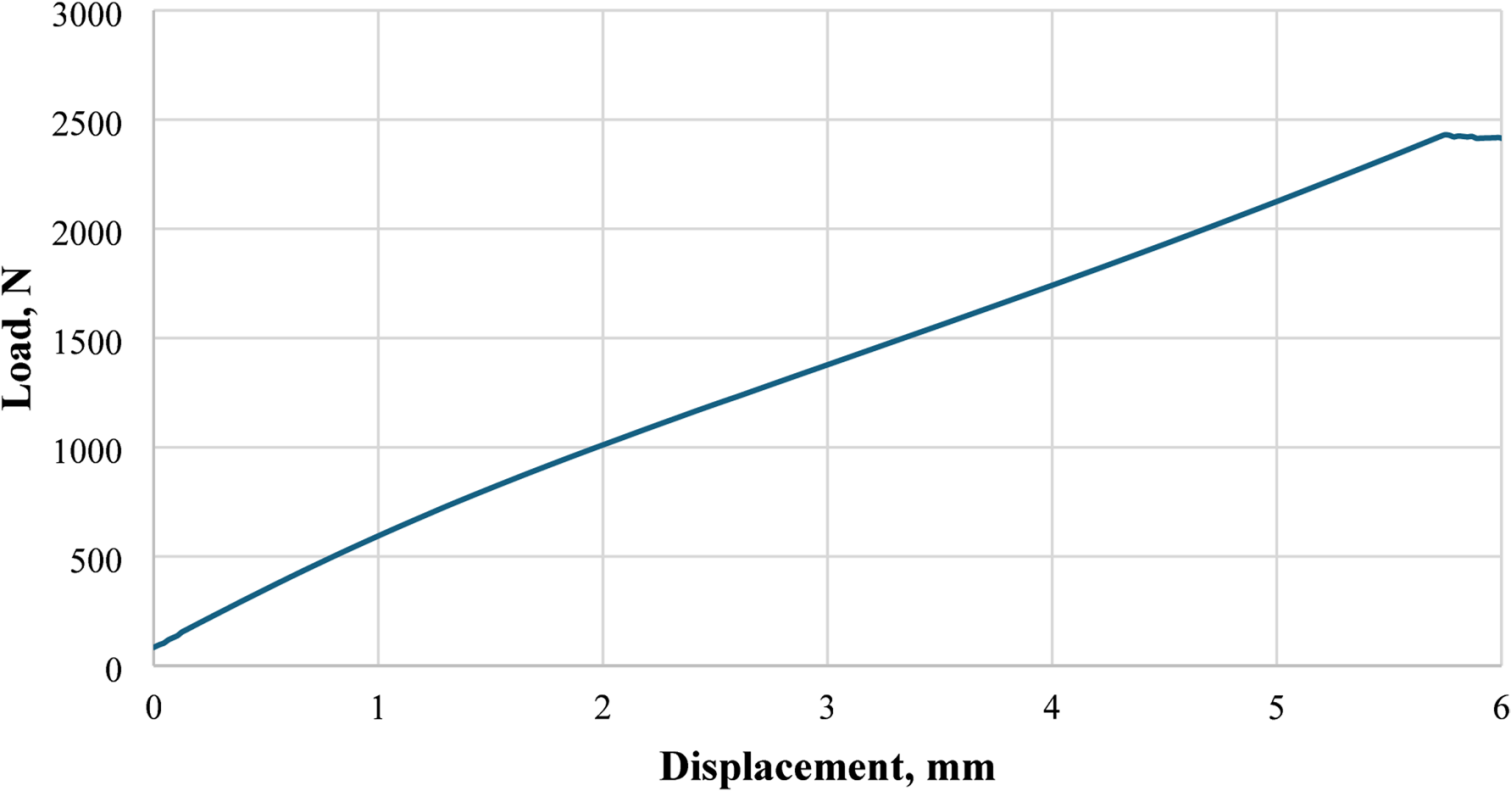
Stripper load average values graph.

**Fig. 8:**
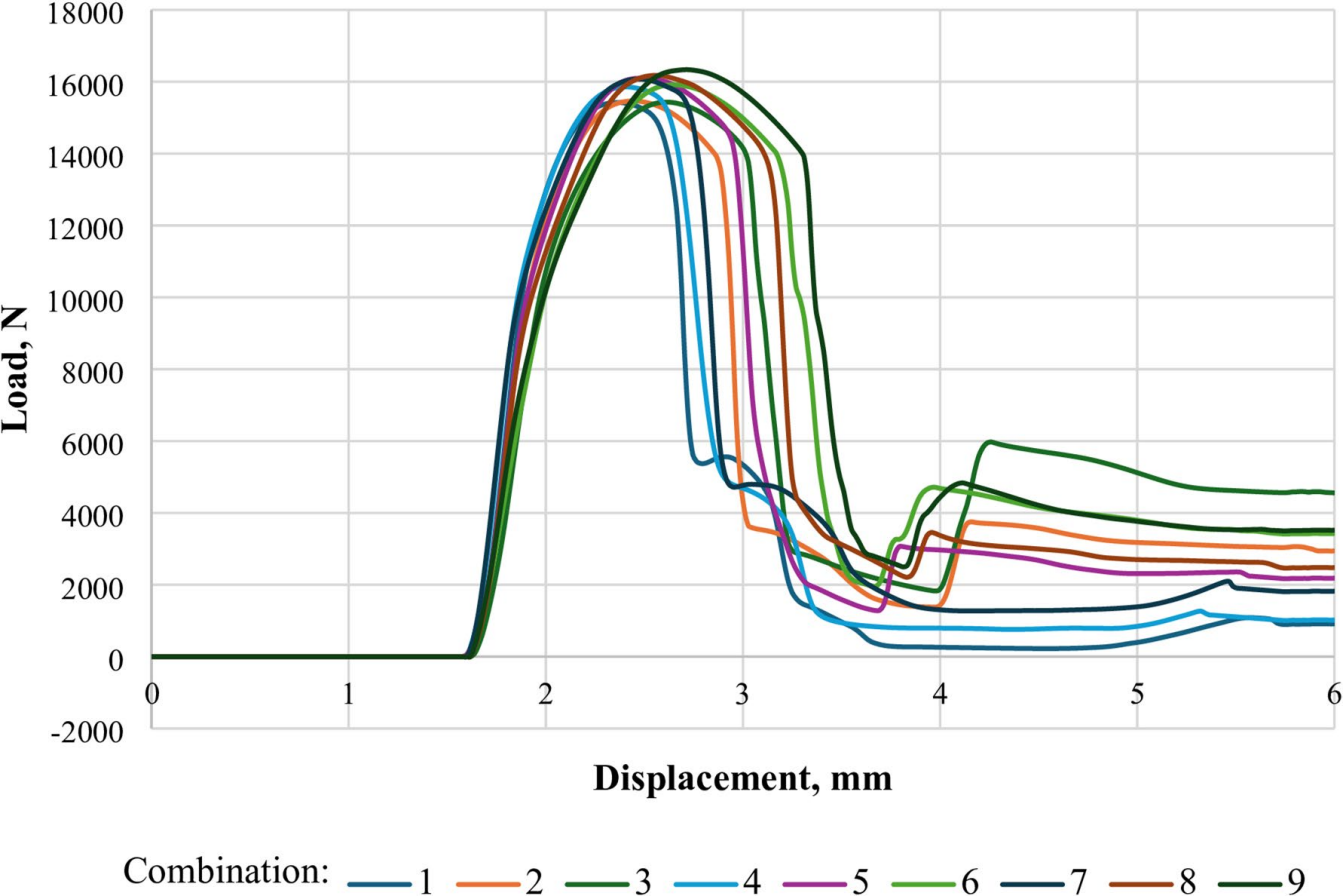
All combination average values graphs without the stripper constituent.

As can be seen from the graphs presented in Fig. 6, only a noticeable load is generated until the displacement reaches around 1.6 mm, due to the increasing rubber stripper load. From 1.6 mm to approximately 2.5–3.4 mm, the main phase of hole piercing takes place. From 2.8 to approximately 3.2 mm, the secondary phase of hole piercing takes place. After around 3.5 mm, when the hole is already pierced, the graph shows an increase in load due to scrap collision inside the die, left by the previous repetition. To more precisely compare the data and eliminate the stripper constituent from the static test results, was decided. The graph of the stripper load average values was obtained in the same way as in the previous test. The stripper average value graph is presented in Fig. 7, and the static piercing test values comparison without the stripper constituent is presented in Fig. 8.

**Fig. 9:**
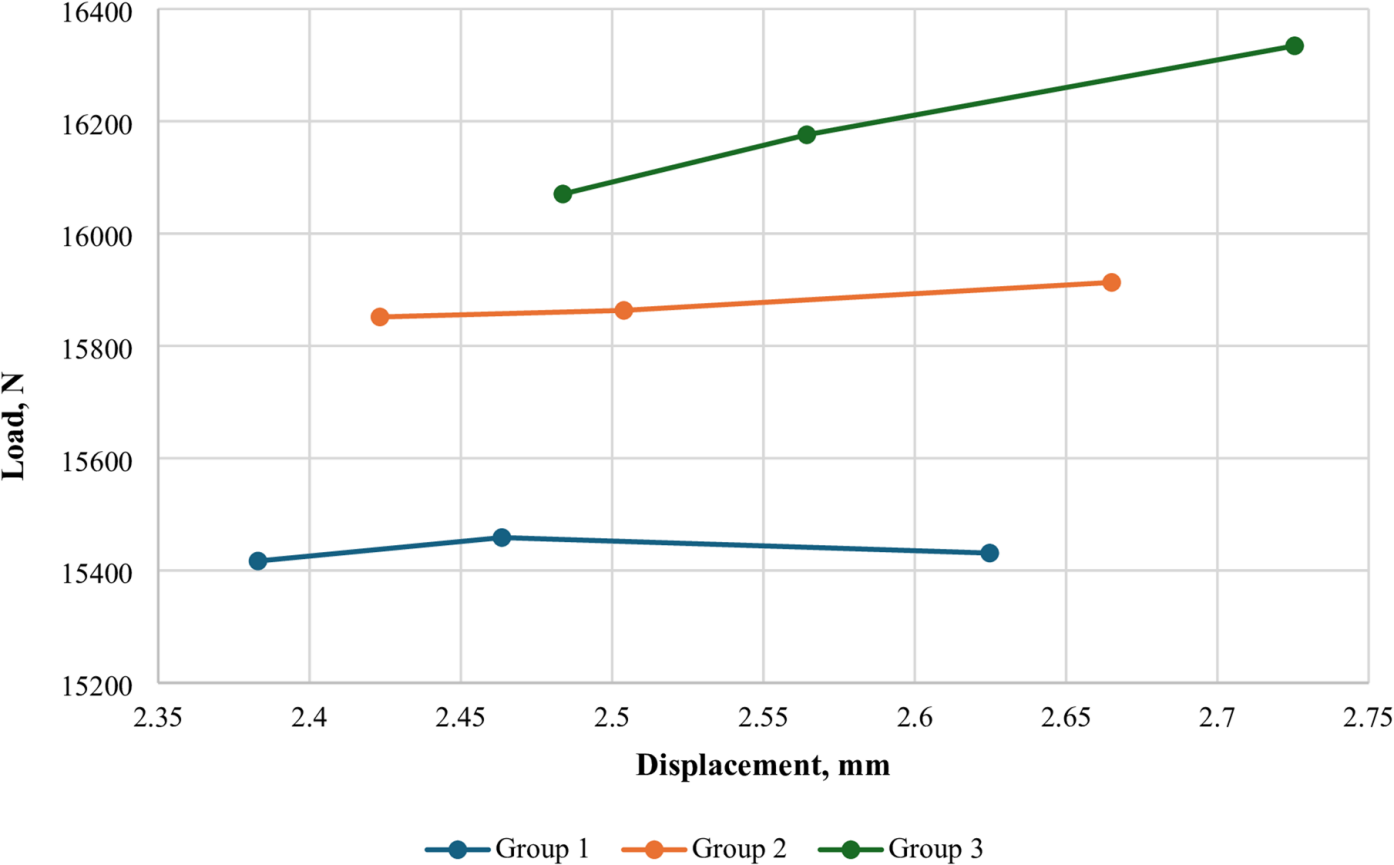
Comparison of static test peak reached load values.

**Fig. 10:**
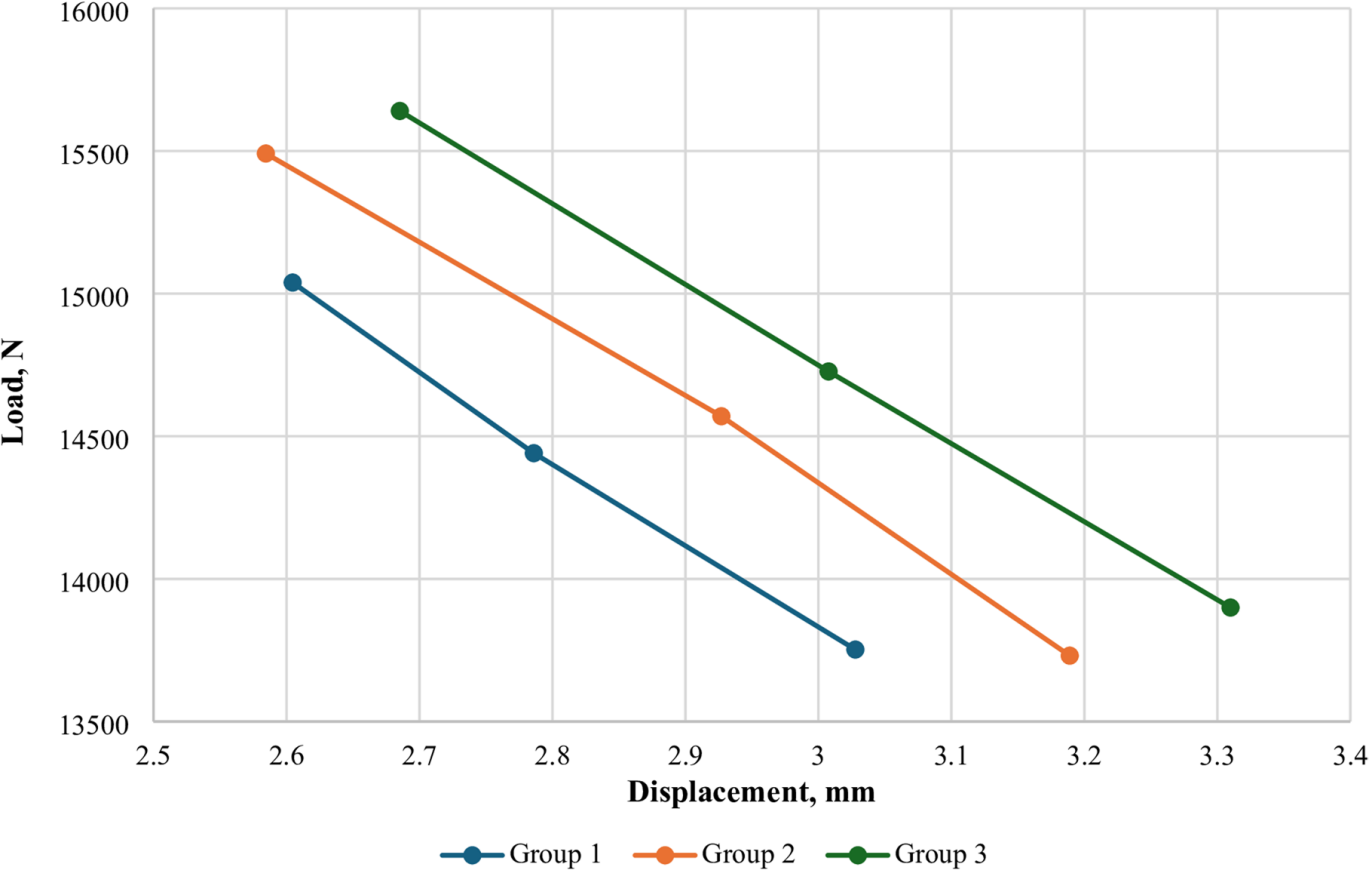
Comparison of static test material breakage values.

By comparing the acquired values, it was clearly seen that, for every combination, the graph moves to the right, indicating that, under the applied wear model, material punching is replaced by stretching, and the by-product of this process is the appearance of burrs. Static test peak acquired values and material breakage values could be seen in Figs. 9 and 10.

From Fig. 9, it is noticeable that the wear of the punch has a greater influence on the peak piercing load than the wear of the die. The test load increases from 15,146 N when the punch radius is R0.0 mm to 16,070 N when the punch radius is R0.4 mm (when the die radius is R0.0 mm). But when comparing static test material breakage values, it could be seen as an absolutely different situation when die wear has a greater influence than punch wear. The material breakage load decreases from 15,038 N when the die radius is R0.0 mm to 13,752 N when the die radius is R0.4 mm (when the punch radius is R0.0 mm).

Further real-life dynamic testing was carried out using the actual production tool to evaluate the impact of punch wear under operating conditions. The experiment was designed to simulate typical production parameters, starting with DC01 material with a nominal 2 mm thickness, processed at 40 cycles per minute. Lubrication levels were kept consistent, in line with the specifications in the technical documentation accompanying the tool.

**Fig. 11:**
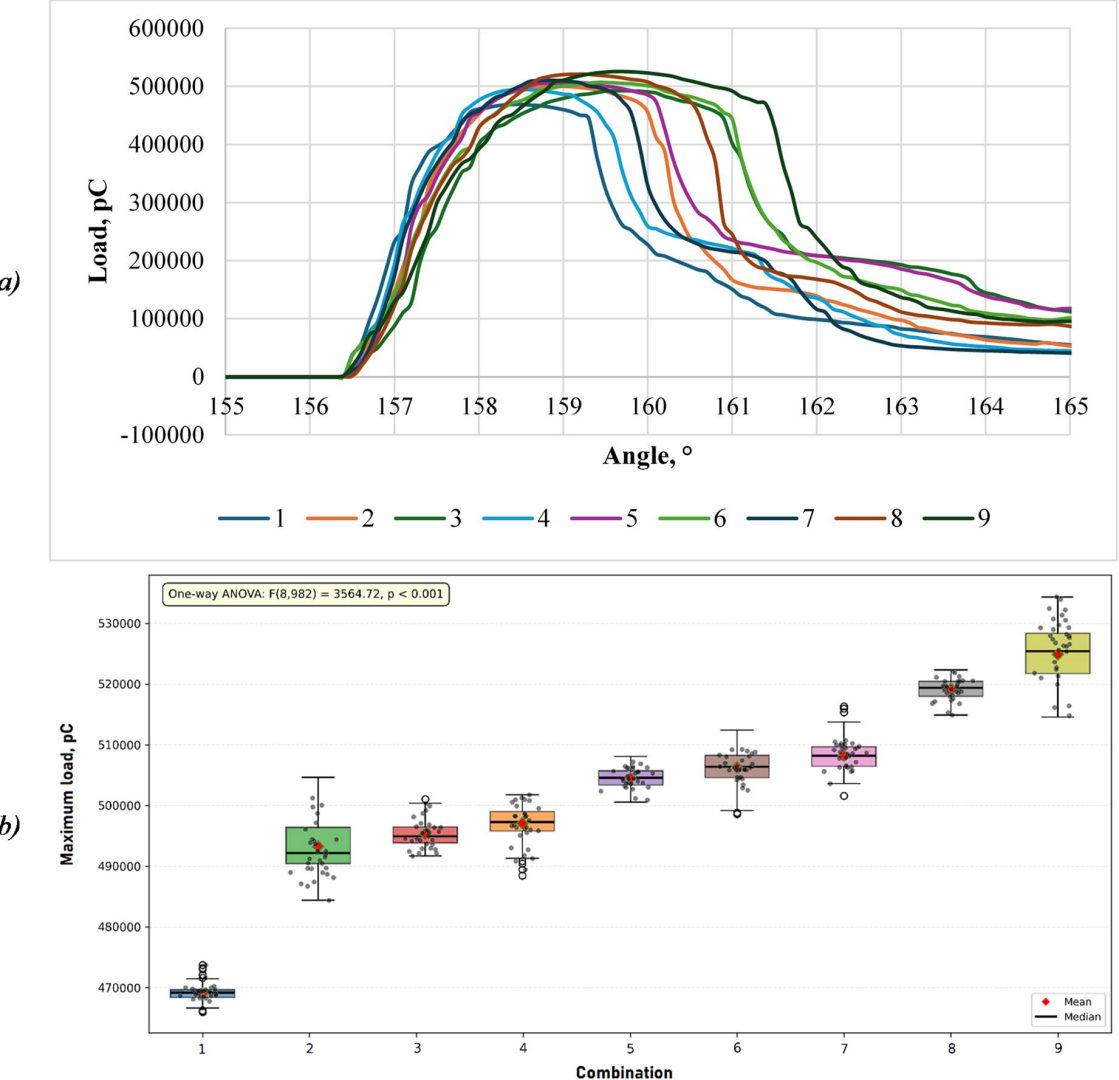
Dynamic tests average load values comparison: (a) Graph view; (b) Recorded values distribution view.

In each case, the tool was subject to between 500 and 550 cycles. This ensured that sufficient data points were obtained to reflect stable processes, disregarding random fluctuations. At the same time, the readings from the piezoelectric sensor were recorded. The average load values obtained from each test condition are presented in Fig. 11.

One of the most noticeable differences between static and dynamic test results is the difference in the shape of the curves. However, this difference is not indicative of a significant difference in the results. The piercing stage in the dynamic tests is completed extremely quickly, within 0.005–0.006 s. This rapid progression is a result of the high-speed motion characteristic of dynamic stamping operations. In contrast, static tests are conducted at much slower speeds, with the piercing stage taking approximately 2.2 s, depending on the material properties and the moment of material breakage.

From a production monitoring perspective, continuously tracking the entire load curve during stamping operations can be challenging. Capturing every detail of the signal curve in real-time is not only complex but may also lead to increased occurrences of unplanned stoppages due to the sensitivity of full-curve monitoring systems to minor fluctuations or noise. Applicable monitoring methods, such as area under the curve and rising slope monitoring, require additional signal processing.

Area under the load curve monitoring represents the total energy consumption accumulated over multiple stages of the piercing process including elastic deformation, fracture, and post-piercing contact. Although this integral measure provides a convenient global description of the energetics of the process, it essentially combines the contributions of mechanisms that are not equally sensitive to the condition of the tool. In particular, the early stage of the curve is dominated by elastic and initial plastic deformation, which is largely governed by material properties and sheet thickness, whereas the fracture stage is more directly influenced by tool sharpness and clearance conditions. Following material separation, the post-peak region often includes continued punch travel, frictional sliding, and possible contact with scrap left from previous step, introducing additional energy components that are weakly correlated with wear progression. As a result, the total area under the curve may exhibit reduced sensitivity to wear specific changes, especially when variations in material behavior or lubrication conditions are present. Moreover, the post-peak segment is prone to measurement noise, signal oscillations, and machine compliance effects, which can disproportionately affect the integrated energy value.

The rising slope method, which estimates the initial gradient of the load displacement curve, is often used to characterize the early-stage contact conditions between the punch and the workpiece. This region primarily reflects elastic deformation and the onset of plastic yielding and therefore can be sensitive to changes in contact stiffness and interface conditions. In principle, sharper tools can produce a steeper initial slope due to more localized stress concentration, while worn tools with rounded edges can show a more gradual increase in load. However, in practice, the rising slope is greatly influenced by system-level factors that are not limited to the condition of the tool. Machine flexibility, stiffness of the press frame and tool assembly can significantly change the measured slope by causing additional elastic deformation unrelated to the cutting interface. Similarly, setup conditions such as alignment, clamping stiffness, lubrication and sensor placement contribute to variability in the early part of the curve. Even small differences in workpiece position or surface conditions can affect the initial contact behavior, further complicating wear level interpretation.

**Fig. 12:**
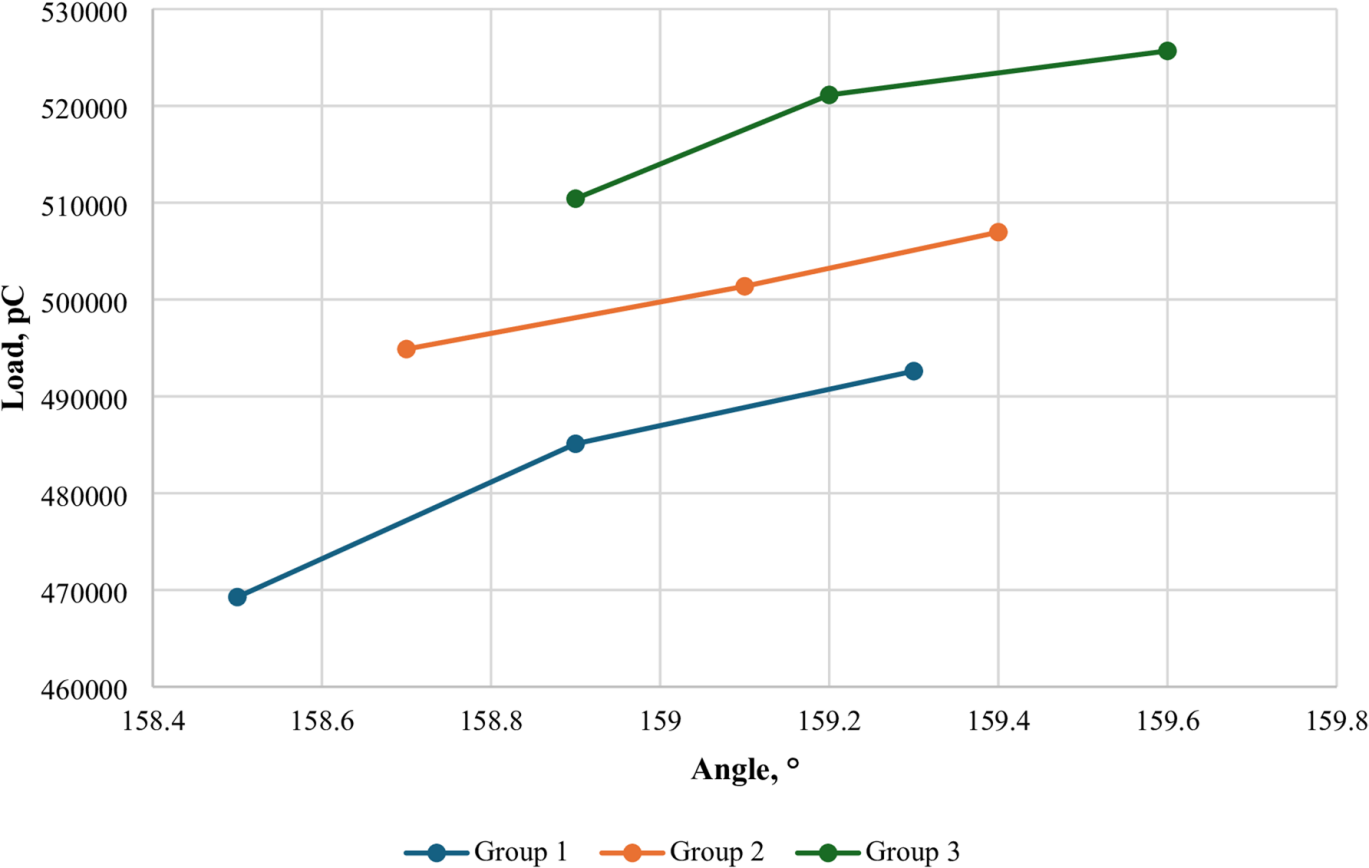
Comparison of dynamic tests’ peak load values.

A more practical and efficient approach in production is to monitor a specific point on the load curve - primarily the peak load value. The peak load directly reflects the instantaneous resistance of the material-tool system from the initial contact moment, when friction, material hardening and edge degradation occur simultaneously. Therefore, for reasons of industrial real-time performance, robustness, and application convenience, the peak value was selected as the main monitoring indicator. The peak load values recorded during separate dynamic tests are summarized and presented in Fig. 12.

To improve the comparison, a quantitative sensitivity analysis was conducted. The relative increase in force and signal peak with respect to tool edge radius was calculated for static and dynamic tests. The resulting trend in both cases was consistent and monotonic. Furthermore, a linear approximation of the relationship between data peaks and edge radius was used, and comparable slopes were observed under static and dynamic conditions. This confirms that the two test sets exhibit similar wear-related behavior despite differences in loading rate.

Based on the assumption that the peak force value during the stamping process is the most sensitive indicator of component wear, a logical approach is to directly compare the results of both static and dynamic tests. By analyzing the form and trend of the load increase over time, it becomes possible to observe how tool wear impacts the process under both testing conditions. Specifically, in both static and dynamic tests, using a sharp die combined with a progressively increasing radius on the piercing punch, a continuous increase in peak values is evident. This consistent increase strongly suggests a direct correlation between rising load levels and the progressive wear of stamping components, particularly the punch and die cutting edges. As wear advances, the process requires more load to achieve the same material separation, validating the initial assumption. A second noteworthy observation concerns the shift in the position of the load peak over the process timeline. Indeed, as the radius of the punch used for piercing increases, the position of the point at which the peak load is reached gradually shifts to the right along the process timeline.

This shift indicates a significant change in the material deformation mechanism. Rather than being cleanly cut by the sharp punch edge, the material increasingly undergoes plastic deformation and stretching before fracturing. This ongoing stretching results in changes to the material’s internal structure and the appearance of defects, which could weaken or reduce the lifetime of the produced part.

These observations highlight the need for the monitoring of the load peak in the stamping process, which is a reliable measure for the detection of tool wear and changes in the cutting mechanism during the stamping process, thereby helping in the predictive maintenance of the system and the reduction of downtime due to excessive wear of the components.

### Wear effects on mechanical and microstructural quality, the SEM observations

After performing tests, it was decided to select combinations 1, 5, and 9 for micro-structural evaluation using the SEM microscope. The aim of this analysis was to qualitatively examine the variation in the surface and near-surface morphology. These combinations represent low, medium, and high wear states and are based on their ability to capture the gradual, systematic evolution of the force-displacement response across the entire experimental matrix. Combination No. 1 has the lowest peak force and the steepest drop after peak, which corresponds to a relatively sharp tool and minimal wear. In contrast, combination 9 exhibits a significantly higher peak force, a wider peak width, and a more gradual transition after the peak, along with higher residual forces, all of which indicate greater simulated wear and increased frictional interaction. Combination 5 was selected because it lies between these two extremes, exhibiting intermediate peak size, peak width, and post-peak behavior, and is therefore a reasonable evaluation of the average wear condition across the experiment matrix. Importantly, these three cases span the observed range of key signal features, including peak force, peak displacement, and post-penetration response, maintaining consistent trends with the simulated wear. Therefore, they provide a reduced yet representative subset of the full dataset, enabling clearer comparative analysis without losing generality in describing wear-dependent behavior. The noteworthy observation is that SEM analysis in this paper is primarily qualitative and utilized for the comparative evaluation of microstructural changes, rather than quantitative measurement.

**Fig. 13:**
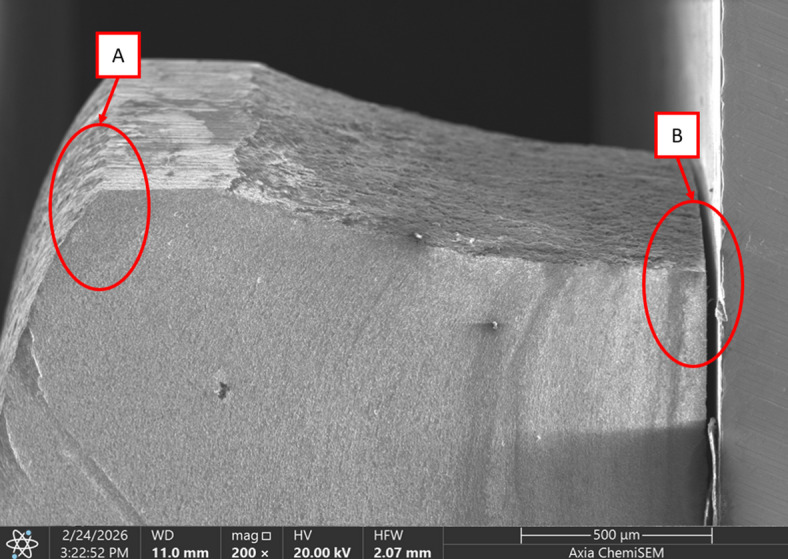
Leftover specimen scanned with SEM: (a) Examined die contact side; (b) Examined punch contact side.

Areas scanned by SEM are presented in Fig. 13, and scanning results are presented in Tables 7 and 8. Materials were collected without metallographic etching, and magnifications ranged up to 1200×.

**Table 7:**
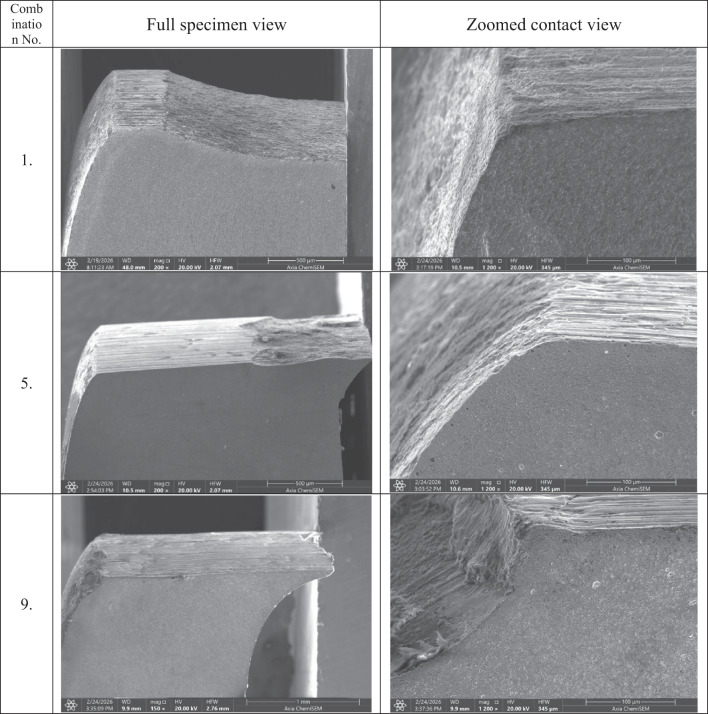
Microscope scanning results on the die contact side.

A trend would appear when defects were matched between all tested specimens on the die contact side. The deformation structure of the reference sample (Combination No. 1) is indicated by the uniform shear zone near the pierced surface that is rather continuous. In the plastically impacted region, aligned lamellar flow lines run parallel to the edge, suggesting that material extrusion is kept stable when piercing. This change from the surface layer to the matrix layer is well recognized and gradual, indicating a moderate strain gradient. The matrix area is dense and uniform, showing little sign of microporosity, without any tearing, sharing, or folding of bands. The fracture surface shows plastic flow and micro-void coalescence, without signs of instability, typical of well-controlled ductile fracture. Likewise, Combination No. 5 shows mainly stratified and smooth deformation along the pierced perimeter. In this scenario, the deformed surface layer consists of parallel lamellar flows in the shear direction. This means stable material extrusion with increasing shear under the applied compression. It shows the smooth but distinct transition zone between the heavily deformed surface layer and the bulk material. This reflects a regulated strain gradient in Combination No. 1. The defect structures in this combination are composed predominantly of fine micropores in the bulk material, which may be due to inclusions that lead to nucleation of microvoids. Tearing, delamination, and heavy shearing banding do not seem to be vital. The degree of deformation is considerably more severe and heterogeneous in Combination No. 9, in contrast to the combinations described above. While the material’s shear structure remains in the surface zone, the deformation zone exhibits significant folding, material dragging, and a clear concentration of shear at the corner region. In addition, the high extent of deformation of the lamellae and the presence of distinct shear banding and sudden curvature of the lamellae indicate a substantially higher strain localization and unequal distribution of stresses in the zone of deformation. Another characteristic of the combination of processes leading to failure in this combination is localized tearing and possibly micro-delamination in the zone of deformation. In the bulk of Combination No. 9, a microporosity zone is noted. However, at this combination, interfacial instability is significantly greater. From the defect point of view, Combination No. 1 exhibits more stable plastic flow and fewer structural discontinuities during the deformation process. Nevertheless, in Combinations No. 5 and 9, the findings indicate higher defect severity, particularly high shear band formation, localized tearing, and greater structural heterogeneity. Such features indicate that specimens had higher localized strain and stress concentrations than other samples, leading to higher residual stresses and subsurface damage due to less ideal piercing conditions.

**Table 8:**
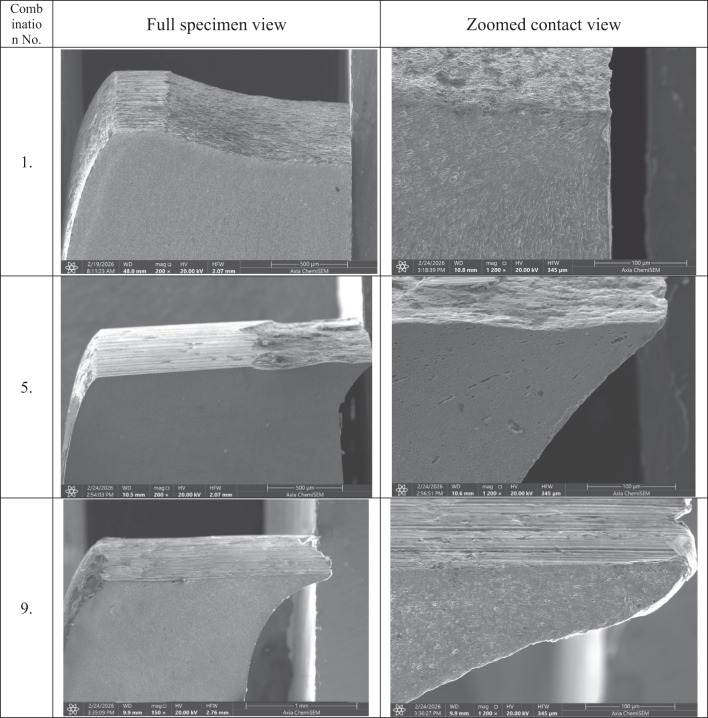
Microscope scanning results on the punch contact side.

The internal structure below the surface of the reference sample (Combination No. 1) exhibits more or less uniform structure. The surfaces of these grains in the topmost layer show plastic deformation, but deeper inside, they’re round and less deformed than those in Combination Nos. 5 and 9. Such a uniform appearance suggests that the local plastic strain is almost negligible and, where any deformation is present, it is spread out relatively more evenly. Combination No. 5, and the material under the surface is relatively uniform and dense with little evidence of major plastic flow. For Combination No. 5 - the microstructure of the material at the top surface level is also comparatively uniform and dense without significant plastic flow. The matrix is equiaxed, with a fine-grained microstructure, little elongation, and no orientation, indicating that plastic strain has not traveled very far. Surface zone-to-material deeper phases transition is gradual, indicating moderate deformation gradients. Above the surface of the upper layer, micro voids and elongated microfeatures containing tensile stresses can form as a result of the piercing process. The uniformity of microfacial features suggests that the material separation was not especially localized and rather distributed across the contact surface. The strong flow lines indicate that it was initially subjected to the compressive stress field with low or no shear-induced grain deformation. Compared to the reference material, the microstructure of Combination No. 5 appears slightly more distorted in the zone immediately below the impacted surface contact layer. For comparison, the area of direct contact shows points of compaction and minor delamination, whereas the material at lower levels exhibits an equiaxed microstructure and negligible elongation, indicating that plastic deformation is concentrated at the surface. Combination No. 9 microstructure: a distinct layered microstructure of material is seen. This implies that the wear simulation showed a high degree of frictional flow interaction within the material. This resulted in consolidating the surface material with high directional plastic flow. The subsurface material beneath the consolidated surface material contains elongated, oriented structures that coalesce into a band aligned in the shear direction. The grains are still somewhat dense in the deeper ground, but micro-voids and inclusions are smaller than the above average. These defects are decreasing and spreading less than the upper level, suggesting that the deformation was dominated by longitudinal and shear flow rather than by the appearance of numerous defects. The area that formed a deformation band aligned towards the surface indicates significant work hardening and stress concentration at the punched edge. Comparison to Combination No. 5 and Combination No. 9 shows that this combination is more structurally aligned and has fewer randomly distributed defects. In contrast, in Combination No. 9, the formation of voids is less statistically significant than plastic flows. This indicates that the piercing was dominated by deep drawing deformation. By comparison, it was found to exhibit more severe plastic deformation and an anisotropic structure, with surface hardening from increased radii on the working edges (wear simulation) during the comparative analysis. With respect to simulated wear levels, this more bulk uniformity is being preserved, primarily limited to the surface and more prominent micro-void features. Thus, the difference in strain and distribution during piercing is expressed here as a more evolved stage of deformation with enhanced surface mechanical strengthening.

## Conclusion

This research demonstrates a strong relationship between stamping force, the condition of the piercing components, and part quality. After conducting research, the following conclusions were formulated:

1. Cutting components’ edge affects the mechanical behavior and microstructural evolution of pierced sheet metal components, as shown by experimental results.
2. Tests show that peak process load increases progressively with increasing edge radius, where in static tests, force increases from 16571.42 N to 17612.73 N, and in dynamic tests, signal strength increases from 468888.92 pC to 524669.59 pC, which indicates that worn tools need more energy to separate the material.
3. Within both tests, the peak of the load shifts toward the process (from 2.38 mm to 2.73 mm in static tests and from 158.5° to 159.6° in dynamic tests), demonstrating the transition from shear-dominated cutting to plastic deformation-dominated fracture.
4. SEM analysis suggests that microstructural damage tends to increase with wear. Sharp tools generate a stable shear zone with minimal subsurface damage, while worn tools tend to form heterogeneous deformation structures, pronounced shear bands, and localized tearing.
5. Greater tool wear may contribute to increased strain localization, structural anisotropy, and residual stress accumulation near the pierced edge, which in turn can deteriorate the mechanical performance as well as fatigue resilience of the stamped components.
6. The proposed monitoring approach enables real-time monitoring of tool degradation and generates a convenient foundation for a predictive maintenance strategy and better process stability in industrial stamping conditions.

## Data Availability

The original contributions presented in this study are included in the article. Further inquiries can be directed to the corresponding author.
